# Current Endovascular Management of Arterial Complications After Pediatric Liver Transplantation in a Tertiary Center

**DOI:** 10.1007/s00270-023-03557-0

**Published:** 2023-10-13

**Authors:** Paolo Marra, Riccardo Muglia, Carlo Alberto Capodaglio, Ludovico Dulcetta, Francesco Saverio Carbone, Naire Sansotta, Domenico Pinelli, Antonio Celestino, Giuseppe Muscogiuri, Ezio Bonanomi, Stefano Fagiuoli, Lorenzo D’Antiga, Michele Colledan, Sandro Sironi

**Affiliations:** 1 School of Medicine and Surgery, University of Milano Bicocca, 20126 Milan, Italy; 2 Department of Radiology, ASST Papa Giovanni XXIII Hospital, Piazza OMS 1, 24127 Bergamo, Italy; 3 Department of Pediatric Hepatology, Gastroenterology, Transplantation, ASST Papa Giovanni XXIII Hospital, 24127 Bergamo, Italy; 4 Department of Organ Failure and Transplantation, ASST Papa Giovanni XXIII Hospital, 24127 Bergamo, Italy; 5 Pediatric Intensive Care Unit, ASST Papa Giovanni XXIII Hospital, 24127 Bergamo, Italy; 6 Gastroenterology, Hepatology and Transplantation Unit, ASST Papa Giovanni XXIII Hospital, 24127 Bergamo, Italy

**Keywords:** Pediatric liver transplantation, Endovascular treatment, Angioplasty, Stenting, Coronary stent-graft

## Abstract

**Purpose:**

Pediatric liver transplant surgery is burdened by arterial complications whose endovascular treatment is not standardized. We report the outcomes of a cohort of pediatric recipients with hepatic artery complications treated by endoluminal procedures.

**Materials and Methods:**

From December 2019 to December 2022, consecutive transplanted pediatric patients who underwent endovascular treatment of hepatic artery complications were reviewed. The analysis included: type of complication (occlusion, stenosis, pseudoaneurysm); onset (acute =  < 15 days, subacute = 15–90 days, late =  > 90 days); endovascular technique (angioplasty, stenting); complications and outcomes. Technical success was defined as the opacification of the hepatic artery at the final angiogram with < 50% residual stenosis and no pseudoaneurysms. Clinical success was defined by graft’s and patient’s survival.

**Results:**

Seventeen patients (8 males; median age 33 months, IQR 9–103) underwent 21 hepatic arteriography procedures for predominantly acute or subacute occlusions (*n* = 7) or stenosis (*n* = 11) with concurrent pseudoaneurysms (*n* = 4). Primary and secondary technical success was achieved in 13/18 and 3/3 procedures, respectively, with overall technical success of 76%. Angioplasty alone was successful in 5/21 procedures; stent-retriever thrombectomy was performed in one occlusion with thrombosis; stenting was required in 9/17 (53%) patients. Clinical success was obtained in 14/17 (82%) patients with hepatic artery patency after a median of 367 days (IQR 114.5–500). Clinical failure occurred in 3 permanent occlusions, with 2 deaths and 1 re-transplantation. Procedure-related complications included minor events in 3/17 (18%) patients and 1/17 (6%) death.

**Conclusion:**

In liver transplanted children with hepatic artery complications, endovascular treatment may provide clinical success, with stenting often required in acute and subacute conditions.

**Level of Evidence:**

Level 4.

**Graphical Abstract:**

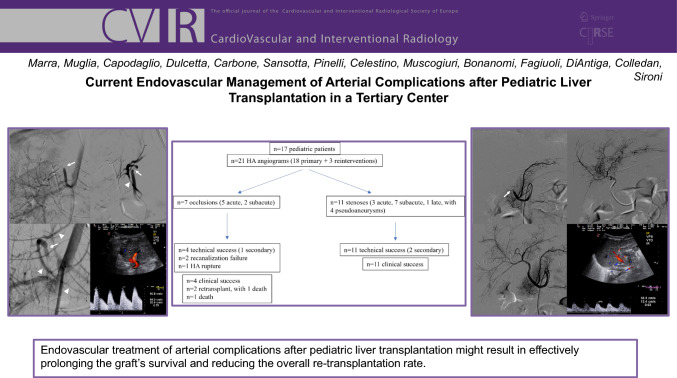

**Supplementary Information:**

The online version contains supplementary material available at 10.1007/s00270-023-03557-0.

## Introduction

Liver transplant (LT) is the treatment of choice in pediatric end-stage liver disease (ESLD). Although advances in surgical techniques and medical management have improved the outcomes, success is still hindered by several complications. One of the main burdens of surgery is the relatively high rate of hepatic artery (HA) complications [1, 2]. Thrombosis is the most common and feared one and can occur in up to 26% of cases, potentially leading to ischemic biliary complications and graft loss [3]; the reported 20-year graft survival for patients not undergoing revascularization is 24%[1]: given the high morbidity and mortality, its timely detection is crucial [4]. Stenosis is reported with an incidence ranging from 4 to 11% in LT patients, and up to 12% in children, but its clinical impact is debated [5]. Pseudoaneurysms are rare but may lead to fatal bleeding [6]. Currently, it has been suggested that endovascular treatment could be an alternative to surgery or re-transplantation even for early-onset complications, as it is less invasive, burdened by lower rates of morbidity and mortality, with similar long-term overall survival [7, 8]. However, it is not universally adopted and techniques are not standardized yet.

The aim of the study is to report the outcomes of the interventional radiological (IR) management of arterial complications after pediatric LT.

## Materials and Methods

### Data Collection

This study was performed in a national tertiary center with more than 20-year experience in pediatric LT surgery. Patients were retrospectively reviewed from a prospectively collected database from December 1st 2019 to December 31st 2022. Consent was obtained from patients’ parents or legal guardians, and the study coded IRMA_POLT was approved by the institutional review board (N. 067/23).

Inclusion criteria were the following: (a) living or deceased-donor LT performed in children (< 18 y.o); (b) availability of follow-up data according to the hospital’s routine for pediatric LT recipients; (c) Computed tomography angiography (CTA)-confirmed diagnosis of HA complications.

### Diagnosis

As per institutional protocol, routine post-LT follow-up was based on color-Doppler ultrasound (CDUS) performed daily the first two weeks after LT, then weekly up to 1 month and every 6–12 months thereafter. During the first year after LT, additional CDUS controls were performed in case of clinical or laboratory alterations. CTA evaluation was performed to confirm CDUS suspicion of vascular complications. Imaging data along with clinical and laboratory findings was discussed within a multidisciplinary team of pediatricians, surgeons and interventional radiologists.

### Type of Arterial Complication

The surgical technique for hepatic artery anastomosis is described in Online Resource 1.

The complications involving the hepatic artery were classified as occlusion, stenosis and pseudoaneurysm [9, 10].

Occlusion (including thrombosis without an underlying obstruction) was defined at CDUS as the absence of flow in the main hepatic artery and intrahepatic branches or by a progressive decrease in systolic and diastolic flow with peak systolic velocity < 30 cm/s. Occlusion was confirmed at CTA as the absence of hepatic artery opacification.

Stenosis was diagnosed at CDUS based on the presence of: *tardus parvus* profile (prolonged systolic acceleration time > 80 ms) and/or anastomotic velocity > 2 m/s and/or intrahepatic velocity < 50 cm/s in association with resistive index (RI) < 0,5. Stenosis was defined at CTA as a focal ≥ 70% narrowing of the hepatic artery. In case of discordance between CDUS and CTA, the clinical management was based on the former.

Pseudoaneurysm was defined as a focal abnormal arterial dilation with irregular morphology regardless of size both at CDUS and CTA.

### Time of Onset

HA complications were also classified according to time of onset from surgery in:

acute if < 15 days; subacute if > 15 days and < 90 days; late if > 90 days.

### Endovascular Technique

All procedures were performed under general anesthesia and were supervised by the same interventional radiologist with more than 5 years of experience, using digital subtraction angiography (DSA) guidance (Allura Xper FD20; Philips Healthcare, the Netherlands). Transfemoral arterial access was established under ultrasound control using a micropunture set (MAK, Merit, USA). A 4-Fr Cobra 2-shaped catheter (Terumo, Japan; Cordis, USA) was placed in the celiac trunk to perform a diagnostic angiogram; the short 4 Fr femoral introducer sheath was exchanged with a longer 5 or 6 Fr sheath (Super Arrow Flex, Teleflex, USA). The use of an intermediate 5 Fr guiding catheter (Envoy, Codman, USA) was considered in case of difficult anatomies. A 0.021–0.027” ID microcatheter (Carnelian, Tokai, Japan; Progreat, Terumo, Japan; Direxion, Boston Scientific, USA; Headway, MicroVention, USA) was navigated in the intrahepatic branches over 0.014–0.018” hydrophilic guidewires (Transend-Fathom, Boston Scientific, USA; Synchro, Stryker Neurovascular, USA). Superselective angiograms were acquired to confirm regular intrahepatic arterial branch catheterization. A 300-cm 0.014” exchange guidewire (Thruway, Boston Scientific, USA; Command, Abbott, USA) was then advanced to support PTA or stent deployment.

If no bleeding occurred, heparin was intra-arterially administered before PTA/stenting by intra-arterial bolus at the dose of 50–80 units/kg. PTA was considered as first-line treatment in case of thrombosis or stenosis without pseudoaneurysm. Coronary monorail (Emerge, Boston Scientific, USA) or peripheral over-the-wire (Sterling, Boston Scientific, USA; Amphirion, Medtronic, USA) balloon microcatheters were used according to the size of the target hepatic artery (range 1.5–5 mm). The neurovascular stent retriever (Solitaire AB, Ev3 Inc, USA) was considered in case of thrombosis after PTA failure. Stenting (coronary Onyx Resolute, Medtronic, USA; coronary Synergy, Boston Scientific, USA; peripheral Herculink, Abbott, USA) or stent grafting (coronary PK Papyrus, Biotronik, UK) was chosen in case of refractory obstructions. Stent grafting was considered in case of pseudoaneurysms and recent surgical anastomosis.

Low-molecular-weight heparin prophylaxis was maintained up to 2 weeks after the procedure. Aspirin was routinely administered for 6 months after transplant at the dosage of 5 mg/kg (up to 100 mg) daily, it was never withheld before the procedure, and indefinitely maintained after revascularization.

### Outcomes and Follow-Up

Technical success was defined according to the type of complication as: regular opacification of the HA at the final angiogram after IR treatment in case of occlusion; residual stenosis ≤ 50% and normalization of CDUS parameters; exclusion of the pseudoaneurysm with regular opacification of the HA. Technical failure was the inability to recanalize the hepatic artery or residual stenosis > 50%. Clinical success was defined graft’s and patient’s survival; clinical failure as the unfavorable development of patient status, resorting to re-transplantation or death. The development of biliary complications was also recorded. Procedure-related complications were classified according to Standard reporting of CIRSE [11].

HA patency was primarily assessed with CDUS with the following timing: immediately after the procedure; within 24 h from the procedure; after 1, 2 and 4 weeks; after which routine follow-up was resumed. CTA was performed only in case of inconclusive findings at CDUS or upon specific clinical indications.

### Statistical Analysis

Descriptive statistics were calculated using SPSS version 26 (IBM, USA). Continuous variables were reported as medians and interquartile ranges, while categorical variables as numbers and percentages. Due to the descriptive nature of the study, it was not lead by any statistical hypothesis.

## Results

**Fig. 1:**
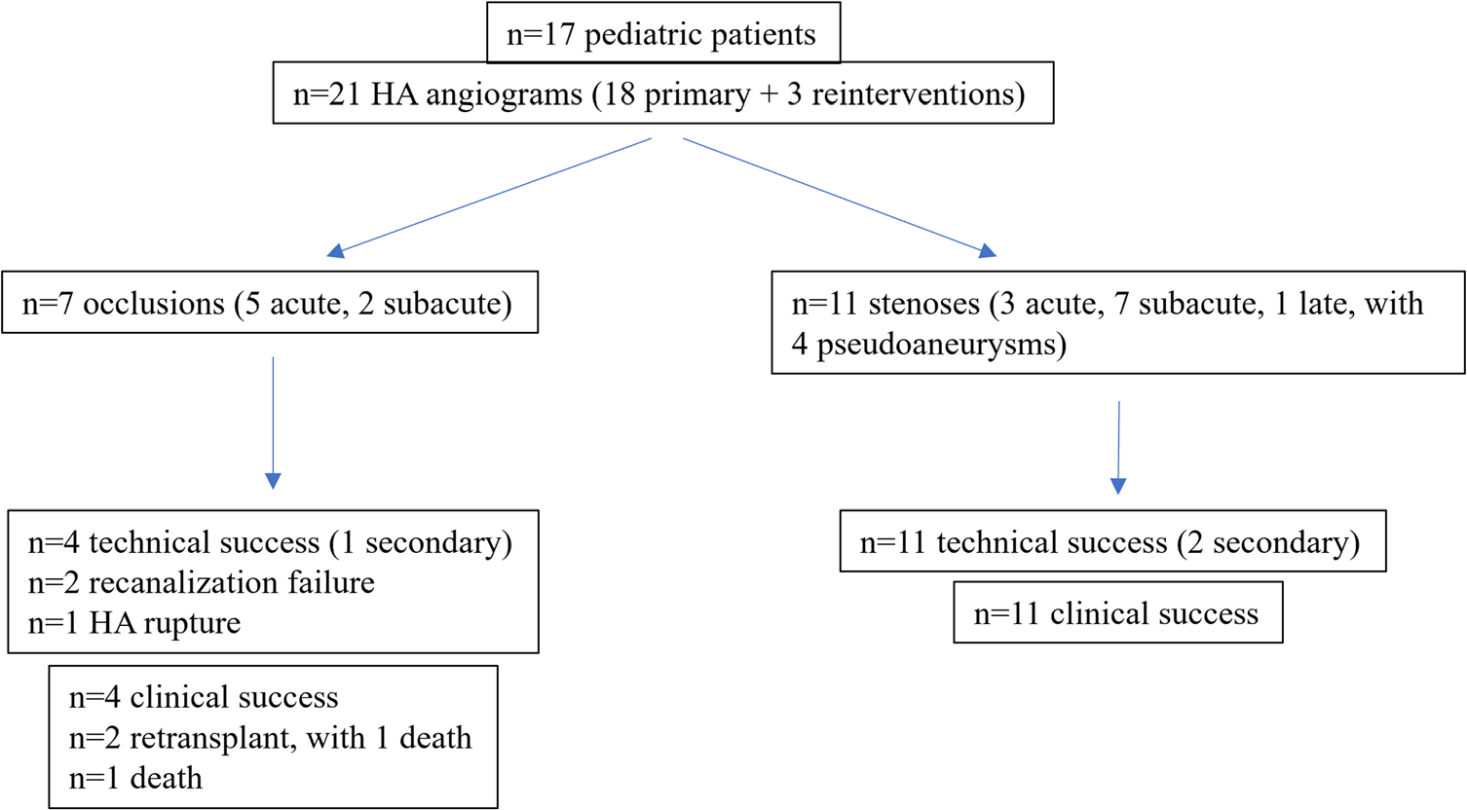
Algorithm of the study population. HA, hepatic artery

In the considered 36-month period, the center performed 86 pediatric LT (73 primary transplants, 13 re-transplants), with 2 living-donor split grafts, 67 deceased-donor split grafts and 17 deceased-donor whole grafts; one meso-Rex bypass surgery was performed in a child with previous split liver transplant complicated by portal vein thrombosis. Out of 86 LT and 1 meso-Rex surgery, 17 (19.7%) patients presented with arterial complications (*M* = 8; median age = 33 months; IQR 9–103 months) and underwent 21 transfemoral endoluminal procedures, detailed in Online Resource 2. Among them, 7 occlusions (5 acute and 2 subacute onset) and 11 stenoses (3 acute, 7 subacute and 1 late onset) with 4 pseudoaneurysms were recorded. More than one interventional procedure was needed in 3 patients; one patient presented complications on both the primary and retransplanted grafts. Figure 1 summarizes the results.

The median time of complication detection from LT (or meso-rex surgery in one case) was 28.5 days (IQR 7–43) and from complications detected at CDUS to CTA was 1 day. The median delay from CTA to arteriography was 0.5 days. In the study period, two further patients with partial hepatic artery occlusion were diagnosed but they were managed conservatively after multidisciplinary evaluation unfavorable to an interventional procedure.

### Technical Success

Overall technical success was reached in 16/21 (76%) procedures, with primary technical success achieved in 13/18 (72%) procedures and secondary technical success in 3/3 (100%) procedures.

**Fig. 2:**
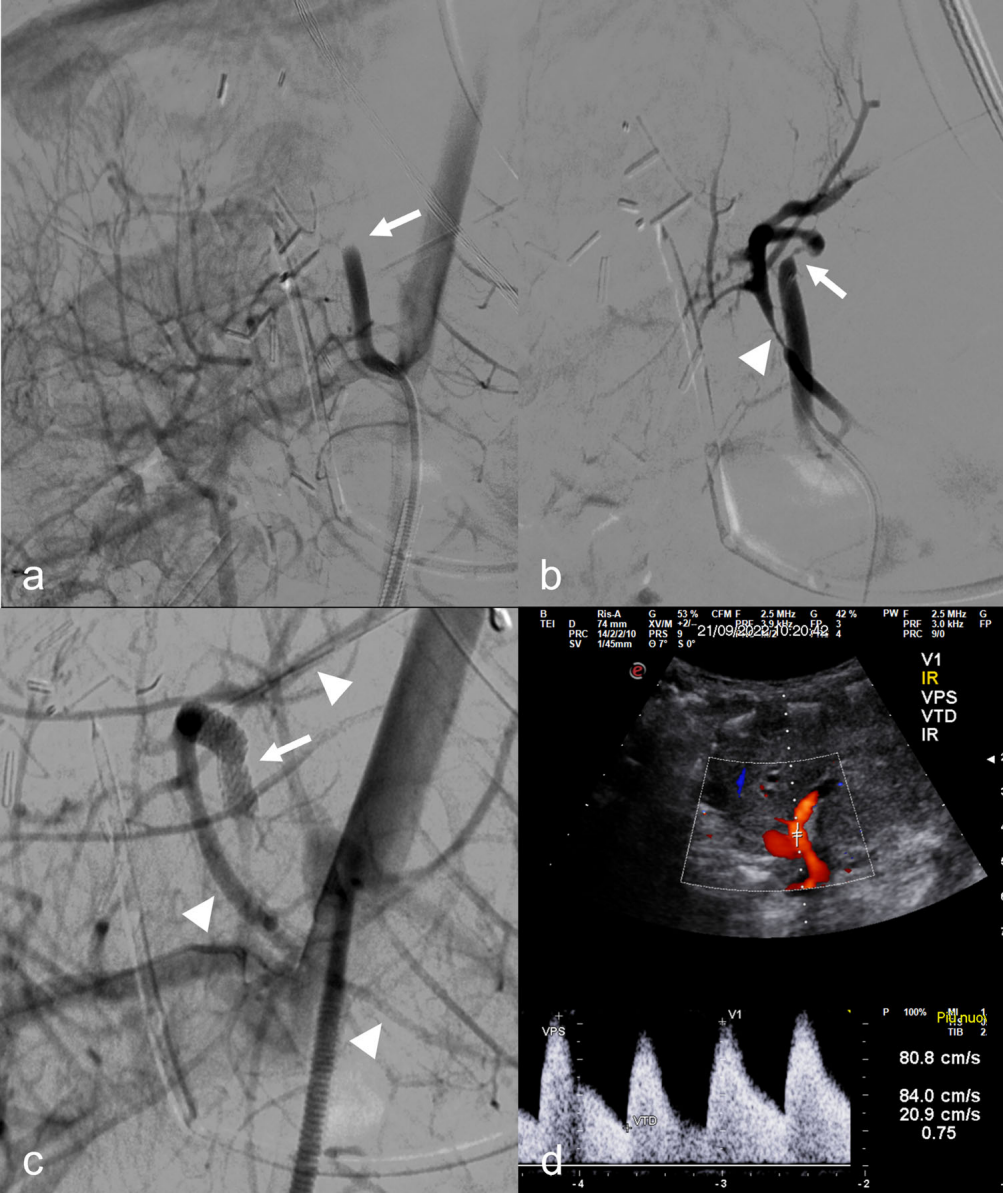
Acute hepatic artery occlusion with underlying kinking and obstruction in a 7-month-old female (case #6) 4 days after split liver transplantation. **a** Selective angiogram of the hepatic artery originating from the superior mesenteric artery shows abrupt occlusion of the vessel (arrow); **b** selective angiogram of the hepatic artery after recanalization shows a kinking and anastomotic stenosis (arrow) as the probable cause of occlusion; also note an intrahepatic spasm (arrowhead) **c** final angiogram performed from the aorta after placement of a coronary stent graft (PK Papyrus 3.5 × 20 mm) shows regular opacification of the hepatic artery and its intrahepatic branches (arrowheads); note spasm disappearance. **d** Follow-up color-Doppler ultrasound shows the patency of the hepatic artery with a normal waveform

**Fig. 3:**
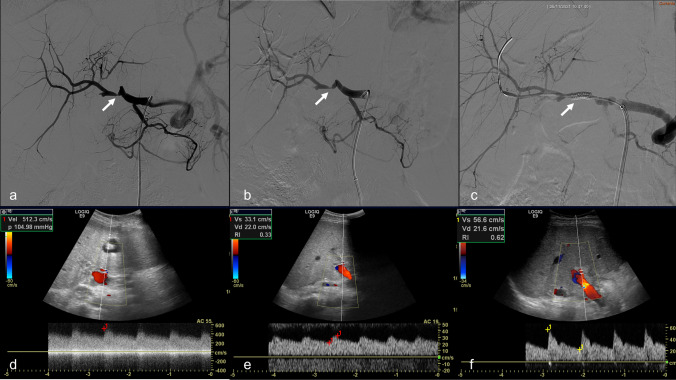
Subacute hepatic artery stenosis in a 14-year-old male (case #14) 30 days after whole liver re-transplantation. **a** Selective angiogram of the hepatic artery shows stenosis of the surgical anastomosis with pre-anastomotic kinking; **b** selective angiogram performed after 3-mm angioplasty shows the persistence of the stenosis; **c** final angiogram performed after placement of a peripheral stent (Herculink 4 × 15 mm) shows resolution of kinking and stenosis and regular opacification of the hepatic artery and its intrahepatic branches. **d** Pre-procedural color-Doppler ultrasound detection of an increased flow velocity at the surgical anastomosis associated with **e** a reduced intrahepatic resistive index (RI = 0.33). **f** Post-procedural color-Doppler ultrasound shows the patency of the hepatic artery with a normalized waveform

**Fig. 4:**
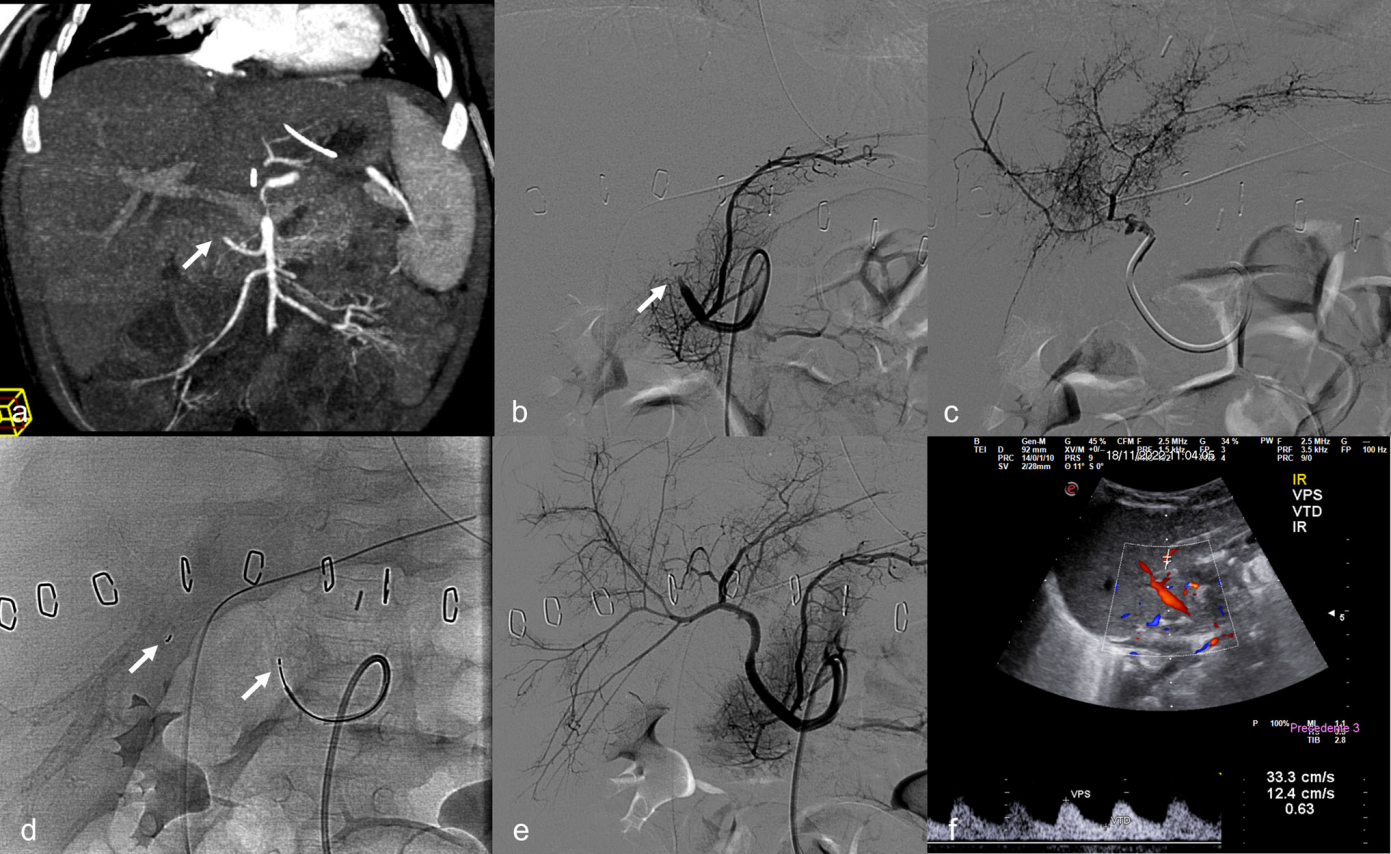
Acute hepatic artery thrombosis in a 8-month-old female (case #10) 7 days after whole liver transplantation. **a** CT-angiography coronal MIP shows abrupt occlusion of the hepatic artery (arrow) originating from the superior mesenteric artery; **b** selective angiogram of the hepatic artery confirms the occlusion at the surgical anastomosis (arrow); **c** selective angiogram performed after recanalization of the hepatic artery with a 4-F catheter shows a very poor intrahepatic arterial circulation; **d** X-ray image acquired during stent-retriever thrombectomy: the arrows indicate the markers at the extremities of the Solitaire AB device; **e** final angiogram of the hepatic artery shows restored opacification of the hepatic artery and its main intrahepatic branches. **d** Follow-up color-Doppler ultrasound shows the patency of the hepatic artery with a normal waveform

Angioplasty alone was successful in 5/21 (24%) procedures; in all the other successful HA recanalization, stenting was necessary in 9/17 (53%) patients and 10/21 (48%) procedures to obtain technical success, due to persistent arterial occlusion, residual stenosis > 50% not responsive to angioplasty or recent surgical anastomosis judged at risk of rupture (Fig. 2). Coronary balloon-expanding devices were used in all but one stenting procedure in which a peripheral bare metal balloon-expanding stent was deployed (Fig. 3). Uncovered coronary stents were used in 2 patients, coronary stent grafts in 5 patients, while both were employed in 1 patient. In one acute occlusion due to thrombosis without an underlying stenosis, successful recanalization of the target HA was achieved by means of mechanical thrombectomy using a neurovascular stent retriever after failed angioplasty (Fig. 4). No patients received thrombolytics. Technical failure occurred in 5/21 (23%) procedures: in one due to arterial kinking that required surgical revision, followed by recurrent occlusion that was managed by endovascular reintervention; in two other occlusions, there was a likely underlying obstruction that couldn’t be crossed; in one procedure, there was a severe stenosis; in the last one, there was a diffuse intrahepatic thrombosis not responsive to angioplasty.

### Clinical Success

Clinical success was achieved in 14/17 (82%) patients. Clinical failure occurred in 3/17 (18%) patients, with 2 deaths due to bleeding and multiorgan failure, respectively, of whom 1 occurred after re-transplantation. Bleeding was a consequence of angioplasty that led to HA rupture. Due to rapid deterioration of the hemodynamic status, embolization was preferred over stent grafting. One patient underwent successful re-transplantation. Online Resource 3 shows transaminase and lactate dehydrogenase values immediately before and within 24 h after the procedure. A mild alteration of pre-procedural liver enzymes was observed in stenosis without a post-procedural increase; liver enzymes were significantly altered in HA occlusions with a relevant post-procedural increase in failed or complicated procedures.

In 14/17 (82%) survived and non-retransplanted patients, regular intrahepatic arterial vascularization was documented with routine post-transplant imaging follow-up after a median of 367 days (IQR 114.5–500).

Procedure-related grade 3 complications occurred in 3 patients: 2 common femoral artery occlusion related to 5 Fr sheath placement, which were conservatively managed thanks to spontaneous limb revascularization through collaterals. One dissection and occlusion of the common HA was compensated through the gastroduodenal artery. One grade 6 complication involved HA rupture leading to shock, multiorgan failure and death.

Out of 17 patients, 10 (59%) developed biliary obstructive complications, without any association with the technical and clinical success of the IR procedures.

## Discussion

We reported our most recent experience in the management of HA complications in pediatric LT recipients with the aim to support IR management, which has become the first treatment approach in our center. HA thrombosis is the most feared complication of liver transplantation, potentially leading to biliary necrosis, sepsis and death, especially when left untreated for more than 8 h [12, 13]. HA thrombosis seems to be age-dependent, with a high prevalence in the age range between 0 and 5 years [14]. Routine imaging follow-up after LT is crucial and aims to promptly detect vascular complications. CDUS is the primary and most easily available modality, as it is easily repeatable, sensitive, cost-effective [9, 15] and provides real-time information on the blood flow [16, 17]. A routine CDUS follow-up protocol is not standardized, varying from centers that perform CDUS only in case of clinical alterations after the second post-transplant day [18] to centers that rely on CDUS every 12 h [8]. A prompt integration of functional CDUS data with morphological information provided by CTA is fundamental to characterize vascular anatomy. The importance of including CTA in the diagnostic algorithm was confirmed in our experience by the incidental detection of pseudoaneurysms, which are rare but potentially catastrophic complications of LT [7, 19]. The high diagnostic value and the short examination time of CTA overweight the risk related to iodinated contrast, ionizing radiations, and the need to sedate pediatric patients [20]. Some research suggested that safe conservative management of asymptomatic HA occlusion is possible [21], thanks to neoangiogenesis, which could permit a long-term survival, although in turn favoring the development of chronic thrombosis, especially in the pediatric population [22]. Nevertheless, the long-term low survival rate in pediatric LT recipients in which neoangiogenesis is unsuccessful justifies the need for urgent revascularization [1]. Unfortunately, the development of biliary complications is unpredictably related to ischemic liver injury, as they occurred also in promptly managed patients in our series, being probably the result of a multifactorial process.

Treatment strategies for HA thrombosis vary according to clinical conditions and local expertise and no standard of care exists. Surgery still plays a major role in many centers with arterial anastomosis revision or re-transplantation [1, 23]. However, early percutaneous revascularization could not only prevent re-transplantation but also serve as a bridge to retransplant: untreated HA thrombosis leads to retransplant in 25–83% of cases, while the rate drops to 28–35% when revascularization has been performed [24, 25]. The null rate of re-transplantation performed after successful percutaneous revascularization of HA occlusion in our series supports the primary role of endovascular treatment. Few retrospective studies reported the long-term outcomes of the endovascular treatment of vascular complications after pediatric LT. Techniques such as PTA, stenting, thrombolysis and mechanical thrombectomy have shown encouraging results [26–29]. Notably, the use of the neurovascular stent-retriever device to treat HA thrombosis after LT has been already reported in adults [30]. We never resorted to intra-arterial thrombolysis in our recent experience, although it may have a success rate of up to 68% [27]. We judge intra-arterial thrombolysis too risky to be performed in young patients and in acute settings. Bleeding complications after intra-arterial thrombolysis are reported in a considerable proportion of cases, leading to fatal abdominal hemorrhage [27]. In the acute setting (up to 2 weeks after transplant), we were also concerned about the potential damage to the recent surgical anastomosis. Therefore, we used undersized low-profile balloon catheters and coronary stent grafts for primary stenting, especially when an underlying obstruction was detected. In subacute and chronic conditions, we relied on angioplasty, with stenting reserved to refractory cases. As one may argue, we were not concerned about the small coronary stent diameters that may be considered undersized with patients’ growth. Indeed, if arterial flow deprivation is not a sudden event, angiogenesis gradually promotes supply of the intrahepatic circulation from different routes. However, repeated angioplasty and stent post-dilation is possible in the eventuality of intrastent stenosis. Long-term data will provide us with this relevant information.

**Fig. 5:**
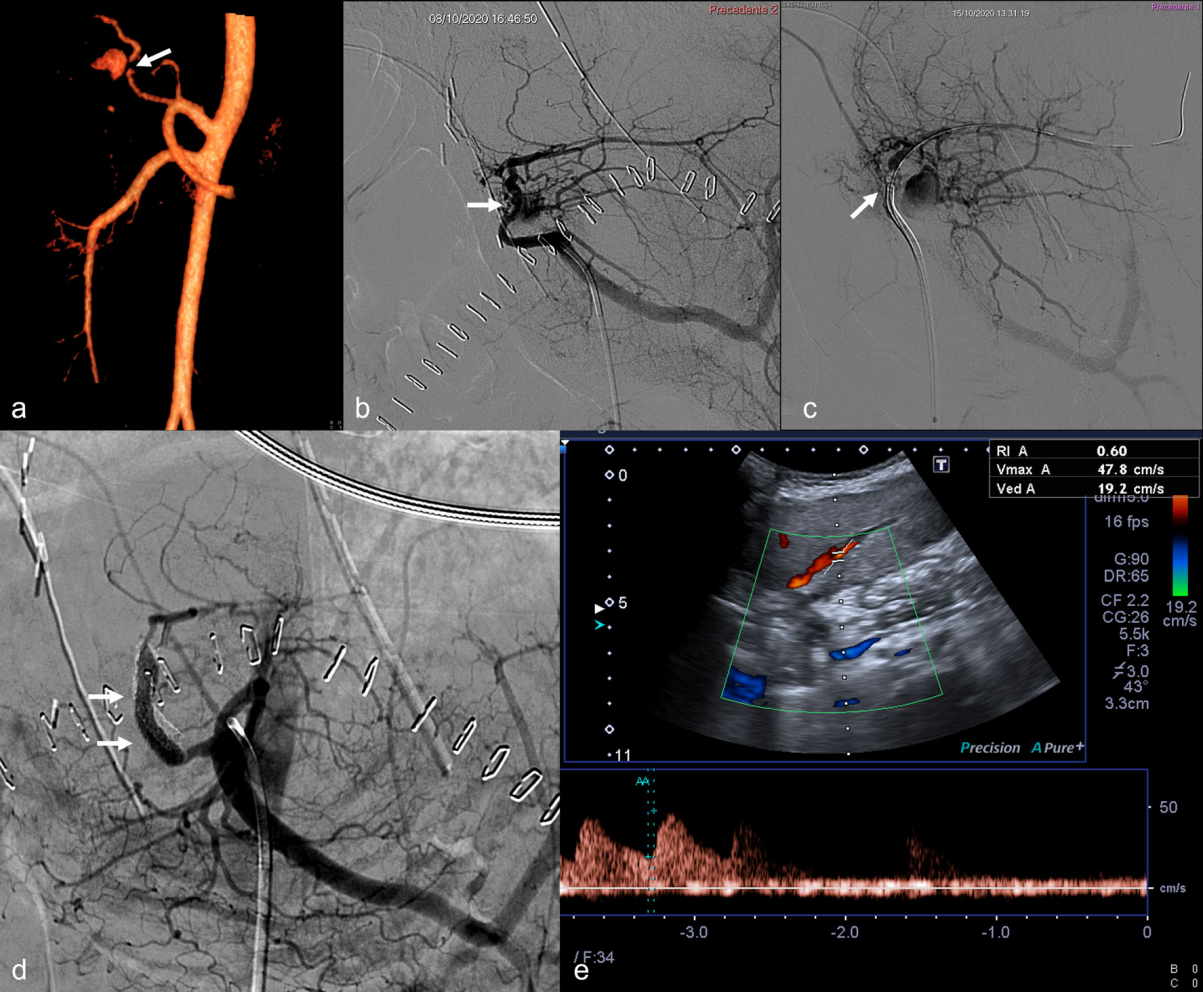
Acute hepatic artery stenosis and pseudoaneurysm in a 3-year-old female (case #7) 2 days after meso-rex bypass creation due to extrahepatic portal vein thrombosis after split liver transplantation. **a** CT-angiography 3D rendering depicts the anatomy of the hepatic artery with stenosis (arrow) and pseudoaneurysm located within the liver. **b** Selective angiogram of the hepatic artery shows the pseudoaneurysm (arrow) involving the origin of the arterial branch for segment 3; **c** selective angiogram performed after placement of the guiding catheter (Envoy 5 F) tip (arrow) across the stenosis and the pseudoaneurysm’s neck; **d** final angiogram performed from the celiac trunk after coaxial stent grafts (arrows, PK Papyrus 3 × 20 mm + 4 × 20 mm) placement shows regular opacification of the hepatic artery and its intrahepatic branches with pseudoaneurysm not opacified. **d** Follow-up color-Doppler ultrasound shows patency of an intrahepatic branch of the hepatic artery with a normal waveform

Stenosis represents a slightly different condition, rarely manifesting as a clinical urgency, with mild or absent clinical and laboratory alterations. Literature reports effective treatment of stenosis of the HA in adult LT patients [31]. The decision between PTA alone or stenting should be based on features like morphology of the stenosis, tortuosity and kinking of the arteries, reserving PTA for shorter and focal stenoses [8, 13, 32, 33]. A recent retrospective study [34] showed comparable outcomes of PTA with or without stenting, with slightly higher stenosis relapse after PTA alone. However, no robust evidence supports primary stenting versus PTA alone, nor superiority of the operative approach versus the conservative one for asymptomatic or late-onset stenosis. A recent retrospective analysis performed on adult liver transplants [35] showed that patients treated for symptomatic stenosis had a longer biliary stricture-free survival compared to conservatively managed patients, although without statistical significance. Moreover, asymptomatic and late-onset stenosis patients that did not receive an endovascular treatment did not develop any biliary stricture. This data supports the hypothesis of collateral vessels suppling the intrahepatic circulation in non-acute-onset obstructions. Although we never considered stenting as the first option, it was frequently performed in our series of stenosis due to failure of simple angioplasty or the presence of pseudoaneurysms. In particular, pseudoaneurysm management with stent graft allowed to preserve HA vascularization (Fig. 5). Even though HA stent grafting to treat pseudoaneurysm is feasible as already reported [36, 37], embolization may be considered an alternative in unfavorable situations.

Our experience differs from the above-mentioned reports: first, our study population was totally pediatric with a median age < 3 years; second, in our series, LT was performed from deceased-donors in all but one case and many were re-transplants; third, HA occlusion and stenosis were predominantly acute or subacute, with just one case of late onset. Despite all these factors, the incidence of arterial complications in our series is consistent with literature [3].

Complications of endovascular treatments may occur in up to 20% of cases [26]. They include thrombosis, intimal dissection, arterial rupture and pseudoaneurysm. In our series, procedure-related complications were conservatively managed in the majority of cases, while arterial rupture led to hemorrhagic shock and multiorgan failure with death in one very fragile patient. As expected, liver enzymes markedly increased after failed or complicated interventions for HA occlusion while kept substantially unchanged in straightforward procedures.

Regarding antithrombotic and anticoagulation prophylaxis, variability exists in clinical practice among centers [31]. Our study cohort was treated homogeneously with a standard protocol.

Limitations of this study include the retrospective analysis restricted to a relatively short and recent period of time with short-term follow-up. If this has provided detailed information favoring consistency of results, diagnostic and interventional technique standardization, the limited number of patients lacking a control group prevents us from a systematic analysis of predisposing factors and from drawing robust conclusions.

## Conclusion

Endovascular treatment of arterial complications after pediatric LT might result in effectively prolonging the graft’s survival and reducing the overall re-transplantation rate. The routine post-transplant follow-up should rely on a close imaging surveillance in the acute and subacute phases and the IR team should be familiar with cutting edge techniques and devices for revascularization therapy.

### Supplementary Information

Below is the link to the electronic supplementary material.

Supplementary file1 (DOCX 15 KB)

Supplementary file2 (DOCX 384 KB)

Supplementary file3 (DOCX 273 KB)

## References

[1] Ackermann O, Branchereau S, Franchi-Abella S, Pariente D, Chevret L, Debray D (2012). The long-term outcome of hepatic artery thrombosis after liver transplantation in children: role of urgent revascularization. Am J Transpl.

[2] Astarcıoglu I, Egeli T, Gulcu A, Ozbilgin M, Agalar C, Cesmeli EB (2019). Vascular complications after liver transplantation. Exp Clin Transp.

[3] Horvat N, Marcelino ASZ, Horvat JV, Yamanari TR, Batista Araújo-Filho JDA, Panizza P (2017). Pediatric liver transplant: techniques and complications. RadioGraph.

[4] Carrillo-Martínez MÁ, Rodríguez-Montalvo C, Flores-Villaba E, Tijerina-Gómez L, Puente-Gallegos FE, Kettenhofen SE (2019). Catheter directed hepatic artery thrombolysis following liver transplantation: Case report and review of the literature. BJR Case Rep.

[5] Orons PD, Sheng R, Zajko AB (1995). Hepatic artery stenosis in liver transplant recipients: prevalence and cholangiographic appearance of associated biliary complications. Am J Roentgenol.

[6] Roberts JH, Mazzariol FS, Frank SJ, Oh SK, Koenigsberg M, Stein MW (2011). Multimodality imaging of normal hepatic transplant vasculature and graft vascular complications. J Clin Imaging Sci.

[7] Gastaca M, Gomez J, Terreros I, Izquierdo J, Ruiz P, Prieto M (2020). Endovascular therapy of arterial complications within the first week after liver transplant. Transpl Proc.

[8] Igus B, Boyvat F, Ozen O, Ayvazoglu Soy EH, Emre K, Haberal M (2022). Role of interventional radiology in the management of early vascular complications after liver transplant. Exp Clin Transp.

[9] Berrocal T, Parrón M, Álvarez-Luque A, Prieto C, Santamaría ML (2006). Pediatric liver transplantation: a pictorial essay of early and late complications. Radiographics.

[10] Thornburg B, Katariya N, Riaz A, Desai K, Hickey R, Lewandowski R (2017). Interventional radiology in the management of the liver transplant patient. Liver Transpl.

[11] Filippiadis DK, Binkert C, Pellerin O, Hoffmann RT, Krajina A, Pereira PL (2017). Cirse quality assurance document and standards for classification of complications: the Cirse classification system. Cardiovasc Intervent Radiol.

[12] Gautier S, Monakhov A, Tsiroulnikova O, Mironkov B, Voskanov M, Dzhanbekov T (2021). Time is of the essence: a single-center experience of hepatic arterial supply impairment management in pediatric liver transplant recipients. Pediatr Transpl.

[13] Saad WEA, Davies MG, Sahler L, Lee DE, Patel NC, Kitanosono T (2005). Hepatic artery stenosis in liver transplant recipients: primary treatment with percutaneous transluminal angioplasty. J Vasc Interv Radiol.

[14] Bezinover D, Deacutis MF, Dalal PG, Moore RP, Stine JG, Wang M (2019). Perioperative thrombotic complications associated with pediatric liver transplantation: a UNOS database evaluation. HPB.

[15] Chiang P-L, Cheng Y-F, Huang T-L, Ou H-Y, Yu C-Y, Hsu H-W (2020). Intensive Doppler ultrasonography for early detection of hepatic artery thrombosis after adult living donor liver transplantation. Ann Transpl.

[16] Sanyal R, Lall CG, Lamba R, Verma S, Shah SN, Tirkes T (2012). Orthotopic liver transplantation: reversible Doppler US findings in the immediate postoperative period. Radiographics.

[17] García-Criado Á, Gilabert R, Berzigotti A, Brú C (2009). Doppler ultrasound findings in the hepatic artery shortly after liver transplantation. Am J Roentgenol.

[18] Seda-Neto J, Antunes da Fonseca E, Pugliese R, Candido HL, Benavides MR, Carballo Afonso R (2016). Twenty years of experience in pediatric living donor liver transplantation. Transplantation.

[19] Pang TCY, Maher R, Gananadha S, Hugh TJ, Samra JS (2014). Peripancreatic pseudoaneurysms: a management-based classification system. Surg Endosc.

[20] Verhagen MV, Dikkers R, de Kleine RH, Kwee TC, van der Doef HPJ, de Haas RJ (2021). Assessment of hepatic artery anatomy in pediatric liver transplant recipients: MR angiography versus CT angiography. Pediatr Transpl.

[21] Fouzas I, Sklavos A, Bismpa K, Paxiadakis I, Antoniadis N, Giakoustidis D (2012). Hepatic artery thrombosis after orthotopic liver transplantation: 3 patients with collateral formation and conservative treatment. Transplant Proc.

[22] Hall TR, McDiarmid SV, Grant EG, Boechat MI, Busuttil RW (1990). False-negative duplex Doppler studies in children with hepatic artery thrombosis after liver transplantation. Am J Roentgenol.

[23] Piardi T (2016). Vascular complications following liver transplantation: a literature review of advances in 2015. World J Hepatol.

[24] Stange B (2003). Hepatic artery thrombosis after adult liver transplantation. Liver Transpl.

[25] Bhattacharjya S, Gunson BK, Mirza DF, Mayer DA, Buckels JAC, McMaster P (2001). Delayed hepatic artery thrombosis in adult orthotopic liver transplantation: a 12-year experience. Transplantation.

[26] Zhu H-K, Zhuang L, Chen C-Z, Ye Z-D, Wang Z-Y, Zhang W (2020). Safety and efficacy of an integrated endovascular treatment strategy for early hepatic artery occlusion after liver transplantation. Hepatobiliary Pancreat Dis Int.

[27] Singhal A, Stokes K, Sebastian A, Wright HI, Kohli V (2010). Endovascular treatment of hepatic artery thrombosis following liver transplantation. Transpl Int.

[28] Peregrin JH, Kováč J, Prchlík M, Heinige P, Kotanová R, Froňek J (2020). Interventional radiological treatment of paediatric liver transplantation complications. Cardiovasc Intervent Radiol.

[29] Sanada Y, Katano T, Hirata Y, Yamada N, Okada N, Ihara Y (2018). Interventional radiology treatment for vascular and biliary complications following pediatric living donor liver transplantation: a retrospective study. Transpl Int.

[30] Meek JC, McDougal JS, Borja-Cacho D, Meek ME (2018). Use of a mechanical thrombectomy device to treat early hepatic artery thrombosis after orthotopic liver transplant. Radiol Case Rep.

[31] Rostambeigi N, Hunter D, Duval S, Chinnakotla S, Golzarian J (2013). Stent placement versus angioplasty for hepatic artery stenosis after liver transplant: a meta-analysis of case series. Eur Radiol.

[32] Molvar C, Ogilvie R, Aggarwal D, Borge M (2019). Transplant hepatic artery stenosis: endovascular treatment and complications. Semin Intervent Radiol.

[33] Ueno T, Jones G, Martin A, Ikegami T, Sanchez EQ, Chinnakotla S (2006). Clinical outcomes from hepatic artery stenting in liver transplantation. Liver Transpl.

[34] Magand N, Coronado JL, Drevon H, Manichon A, Mabrut J, Mohkam K (2019). Primary angioplasty or stenting for hepatic artery stenosis treatment after liver transplantation. Clin Transpl.

[35] Pulitano C, Joseph D, Sandroussi C, Verran D, Strasser SI, Shackel NA (2015). Hepatic artery stenosis after liver transplantation: is endovascular treatment always necessary?. Liver Transpl.

[36] Cui L, Kong L, Bai Y-H, Li X-H, Wang X-Q, Hao J (2020). Covered stent placement for hepatic artery pseudoaneurysm. Abdominal Radiol.

[37] Pedersoli F, Van den Bosch V, Sieben P, Barzakova E, Schulze-Hagen M, Isfort P (2022). Stent graft placement by pseudoaneurysm of the hepatic arteries: efficacy and patency rate in Follow-up. Cardiovasc Intervent Radiol.

