# Supplementary material for: Current Endovascular Management of Arterial Complications After Pediatric Liver Transplantation in a Tertiary Center
Source: Cardiovasc Intervent Radiol. 2023 Oct 13;46(11):1610–20. doi: 10.1007/s00270-023-03557-0 (PMC10616219; doi:10.1007/s00270-023-03557-0)
Supplement: Supplementary file 1 — Supplementary file1 (DOCX 15 KB) [file 270_2023_3557_MOESM1_ESM.docx]

Journal

Cardiovascular and Interventional Radiology

Title

CURRENT ENDOVASCULAR MANAGEMENT OF ARTERIAL COMPLICATIONS AFTER PEDIATRIC LIVER TRANSPLANTATION IN A TERTIARY CENTER

Author’s list

Paolo Marra^1,2^, M.D. (corresponding author) [pmarra@asst-pg23.it](mailto:pmarra@asst-pg23.it) ORCID Id: 0000-0003-4935-8110

Riccardo Muglia^1,2^, M.D. [rmuglia@asst-pg23.it](mailto:rmuglia@asst-pg23.it)

Carlo Alberto Capodaglio^1,2^, M.D. [carloalberto.capodaglio@gmail.com](mailto:carloalberto.capodaglio@gmail.com)

Ludovico Dulcetta^1,2^, M.D. [l.dulcetta@campus.unimib.it](mailto:l.dulcetta@campus.unimib.it)

Francesco Saverio Carbone^1,2^, M.D. [f.carbone15@campus.unimib.it](mailto:f.carbone15@campus.unimib.it)

Naire Sansotta^3^, M.D. [nsansotta@asst-pg23.it](mailto:nsansotta@asst-pg23.it)

Domenico Pinelli^4^, M.D. [dpinelli@asst-pg23.it](mailto:dpinelli@asst-pg23.it)

Antonio Celestino^1,2^, [a.celestino1@campus.unimib.it](mailto:a.celestino1@campus.unimib.it)

Giuseppe Muscogiuri^2,5^, [g.muscogiuri@gmail.com](mailto:g.muscogiuri@gmail.com)

Ezio Bonanomi^6^, M.D. [ebonanomi@asst-pg23.it](mailto:ebonanomi@asst-pg23.it)

Stefano Fagiuoli^2,7^, M.D., Prof. [sfagiuoli@asst-pg23.it](mailto:sfagiuoli@asst-pg23.it)

Lorenzo D’Antiga^3^, M.D. [ldantiga@asst-pg23.it](mailto:ldantiga@asst-pg23.it)

Michele Colledan^2,4^, M.D., Prof. [mcolledan@asst-pg23.it](mailto:mcolledan@asst-pg23.it)

Sandro Sironi^1,2^, M.D., Prof. [ssironi@asst-pg23.it](mailto:ssironi@asst-pg23.it)

Affiliations

^1^Department of Radiology - Papa Giovanni XXIII Hospital, 24127, Bergamo, Italy

^2^School of Medicine and Surgery, University of Milan-Bicocca, 20126, Milan, Italy

^3^Department of Pediatric Hepatology, Gastroenterology, and Transplantation - Papa Giovanni XXIII Hospital, 24127, Bergamo, Italy

^4^Department of Organ Failure and Transplantation, ASST Papa Giovanni XXIII Hospital, 24127, Bergamo, Italy

^5^Department of Radiology, IRCCS Istituto Auxologico Italiano, San Luca Hospital, 20149, Milan, Italy

^6^Pediatric Intensive Care Unit, ASST Papa Giovanni XXIII Hospital, 24127, Bergamo, Italy

^7^Department of Gastroenterology, Hepatology and Transplantation Unit, ASST Papa Giovanni XXIII Hospital, 24127, Bergamo, Italy

Corresponding author’s address:

Department of Radiology, ASST Papa Giovanni XXIII Hospital

Piazza OMS 1, 24127, Bergamo, Italy.

tel. +390352674359

fax +390352674839

[pmarra@asst-pg23.it](mailto:pmarra@asst-pg23.it)

**Online Resource 1**

*Surgical technique*

Either split liver or whole grafts were transplanted with orthotopic technique. A bilateral subcostal incision is used. Dissection of the portal structures is carried in the hilum near the liver so as to provide maximum length of the hepatic artery, common hepatic duct, and portal vein for subsequent reconstruction. The reconstruction is dependent upon the anatomy and size of the donor and recipient hepatic artery. Anastomosis is done with 6/0 to 9/0 polypropylene suture (Prolene; Pronova), in an end-to-end fashion using loupe magnification (×3.5) and microvascular instruments. The interrupted technique is preferred: three sutures are placed at 120° intervals around the circumference of the arteries to be anastomosed; the three stay sutures are used to rotate the vessels, allowing completion of the anastomosis without catching the opposite wall of the vessel. Alternatively, a continuous suture with backwall first approach (parachute technique) is performed.
