# Supplementary material for: Current Endovascular Management of Arterial Complications After Pediatric Liver Transplantation in a Tertiary Center
Source: Cardiovasc Intervent Radiol. 2023 Oct 13;46(11):1610–20. doi: 10.1007/s00270-023-03557-0 (PMC10616219; doi:10.1007/s00270-023-03557-0)
Supplement: Supplementary file 2 — Supplementary file2 (DOCX 384 KB) [file 270_2023_3557_MOESM2_ESM.docx]

Journal

Cardiovascular and Interventional Radiology

Title

CURRENT ENDOVASCULAR MANAGEMENT OF ARTERIAL COMPLICATIONS AFTER PEDIATRIC LIVER TRANSPLANTATION IN A TERTIARY CENTER

Author’s list

Paolo Marra^1,2^, M.D. (corresponding author) [pmarra@asst-pg23.it](mailto:pmarra@asst-pg23.it) ORCID Id: 0000-0003-4935-8110

Riccardo Muglia^1,2^, M.D. [rmuglia@asst-pg23.it](mailto:rmuglia@asst-pg23.it)

Carlo Alberto Capodaglio^1,2^, M.D. [carloalberto.capodaglio@gmail.com](mailto:carloalberto.capodaglio@gmail.com)

Ludovico Dulcetta^1,2^, M.D. [l.dulcetta@campus.unimib.it](mailto:l.dulcetta@campus.unimib.it)

Francesco Saverio Carbone^1,2^, M.D. [f.carbone15@campus.unimib.it](mailto:f.carbone15@campus.unimib.it)

Naire Sansotta^3^, M.D. [nsansotta@asst-pg23.it](mailto:nsansotta@asst-pg23.it)

Domenico Pinelli^4^, M.D. [dpinelli@asst-pg23.it](mailto:dpinelli@asst-pg23.it)

Antonio Celestino^1,2^, [a.celestino1@campus.unimib.it](mailto:a.celestino1@campus.unimib.it)

Giuseppe Muscogiuri^2,5^, [g.muscogiuri@gmail.com](mailto:g.muscogiuri@gmail.com)

Ezio Bonanomi^6^, M.D. [ebonanomi@asst-pg23.it](mailto:ebonanomi@asst-pg23.it)

Stefano Fagiuoli^2,7^, M.D., Prof. [sfagiuoli@asst-pg23.it](mailto:sfagiuoli@asst-pg23.it)

Lorenzo D’Antiga^3^, M.D. [ldantiga@asst-pg23.it](mailto:ldantiga@asst-pg23.it)

Michele Colledan^2,4^, M.D., Prof. [mcolledan@asst-pg23.it](mailto:mcolledan@asst-pg23.it)

Sandro Sironi^1,2^, M.D., Prof. [ssironi@asst-pg23.it](mailto:ssironi@asst-pg23.it)

Affiliations

^1^Department of Radiology - Papa Giovanni XXIII Hospital, 24127, Bergamo, Italy

^2^School of Medicine and Surgery, University of Milan-Bicocca, 20126, Milan, Italy

^3^Department of Pediatric Hepatology, Gastroenterology, and Transplantation - Papa Giovanni XXIII Hospital, 24127, Bergamo, Italy

^4^Department of Organ Failure and Transplantation, ASST Papa Giovanni XXIII Hospital, 24127, Bergamo, Italy

^5^Department of Radiology, IRCCS Istituto Auxologico Italiano, San Luca Hospital, 20149, Milan, Italy

^6^Pediatric Intensive Care Unit, ASST Papa Giovanni XXIII Hospital, 24127, Bergamo, Italy

^7^Department of Gastroenterology, Hepatology and Transplantation Unit, ASST Papa Giovanni XXIII Hospital, 24127, Bergamo, Italy

Corresponding author’s address:

Department of Radiology, ASST Papa Giovanni XXIII Hospital

Piazza OMS 1, 24127, Bergamo, Italy.

tel. +390352674359

fax +390352674839

[pmarra@asst-pg23.it](mailto:pmarra@asst-pg23.it)


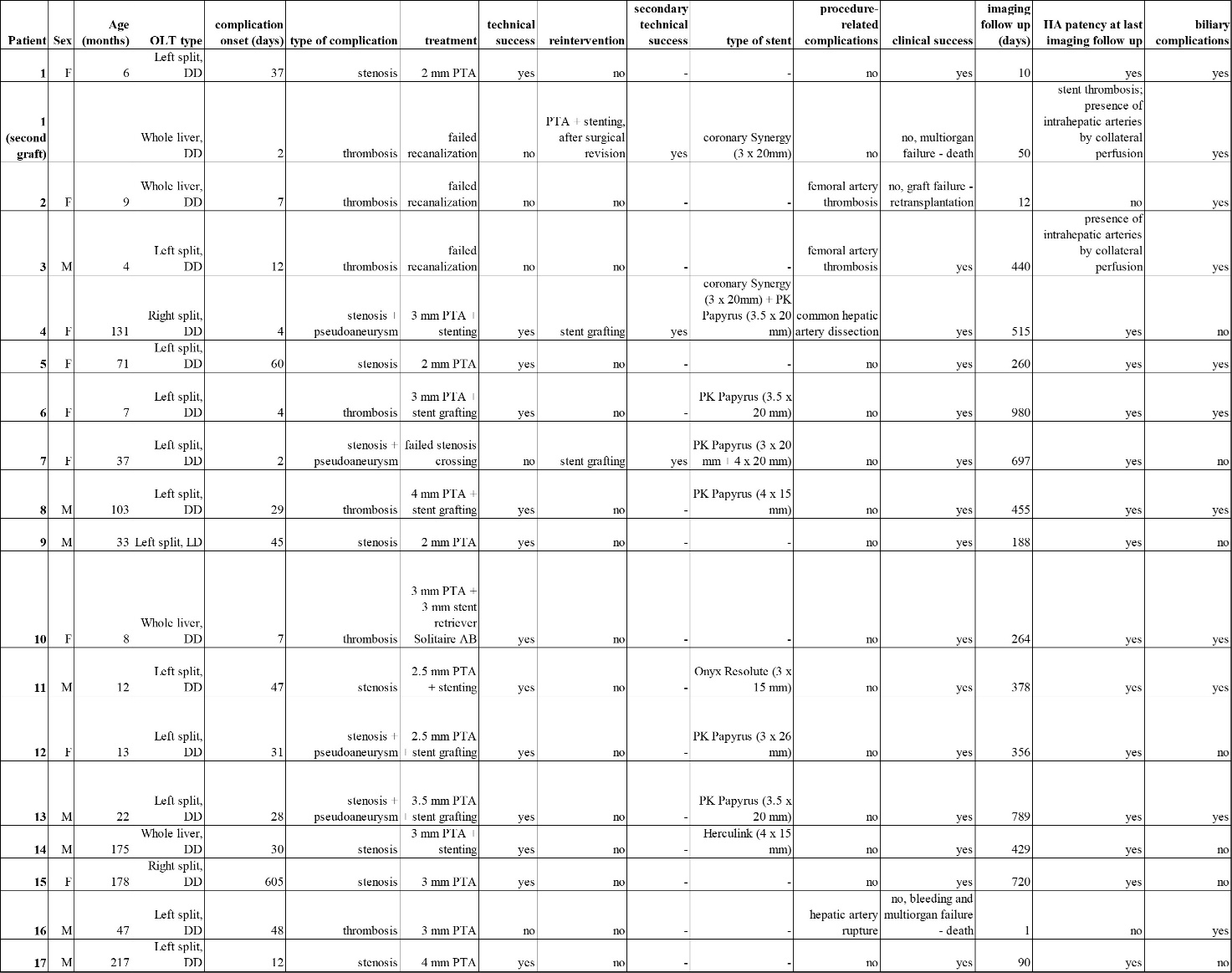


**Online Resource 2** Patients’ characteristics and treatments. BA, biliary atresia; DD, deceased-donor; HA, hepatic artery; LD, living-donor; OLT, orthotopic liver transplant; PTA, percutaneous transluminal angioplasty
